# Phylogeny of the *Varidnaviria* Morphogenesis Module: Congruence and Incongruence With the Tree of Life and Viral Taxonomy

**DOI:** 10.3389/fmicb.2021.704052

**Published:** 2021-07-16

**Authors:** Anthony C. Woo, Morgan Gaia, Julien Guglielmini, Violette Da Cunha, Patrick Forterre

**Affiliations:** ^1^Pôle Analyse de Données UMS 2700 2AD, Muséum National d’Histoire Naturelle, Paris, France; ^2^Département de Microbiologie, Institut Pasteur, Paris, France; ^3^Université Paris-Saclay, CEA, CNRS, Institute for Integrative Biology of the Cell (I2BC), Gif-sur-Yvette, France; ^4^Génomique Métabolique, Génoscope, Institut François Jacob, CEA, CNRS, Univ. Évry, Université Paris-Saclay, Évry, France; ^5^Hub de Bioinformatique et Biostatistique - Département Biologie Computationnelle, Institut Pasteur, Paris, France

**Keywords:** evolution, dsDNA viruses, NCLDV, giant viruses, viral taxonomy

## Abstract

Double-stranded DNA viruses of the realm *Varidnaviria* (formerly PRD1-adenovirus lineage) are characterized by homologous major capsid proteins (MCPs) containing one (kingdom: *Helvetiavirae*) or two β-barrel domains (kingdom: *Bamfordvirae*) known as the jelly roll folds. Most of them also share homologous packaging ATPases (pATPases). Remarkably, *Varidnaviria* infect hosts from the three domains of life, suggesting that these viruses could be very ancient and share a common ancestor. Here, we analyzed the evolutionary history of *Varidnaviria* based on single and concatenated phylogenies of their MCPs and pATPases. We excluded *Adenoviridae* from our analysis as their MCPs and pATPases are too divergent. *Sphaerolipoviridae*, the only family in the kingdom *Helvetiavirae*, exhibit a complex history: their MCPs are very divergent from those of other *Varidnaviria*, as expected, but their pATPases groups them with *Bamfordvirae*. In single and concatenated trees, *Bamfordvirae* infecting archaea were grouped with those infecting bacteria, in contradiction with the cellular tree of life, whereas those infecting eukaryotes were organized into three monophyletic groups: the *Nucleocytoviricota* phylum, formerly known as the Nucleo-Cytoplasmic Large DNA Viruses (NCLDVs), *Lavidaviridae* (virophages) and Polintoviruses. Although our analysis mostly supports the recent classification proposed by the International Committee on Taxonomy of Viruses (ICTV), it also raises questions, such as the validity of the *Adenoviridae* and *Helvetiavirae* ranking. Based on our phylogeny, we discuss current hypotheses on the origin and evolution of *Varidnaviria* and suggest new ones to reconcile the viral and cellular trees.

## Introduction

Studying virus origin and evolution is a challenging exercise, especially when addressing early co-evolution with their cellular hosts. While cellular domains (Archaea, Bacteria, and Eukarya) have been established based on ribosomal RNA sequences and recovered later in many single universal protein trees (Woese et al., 1990), viral “realms” have been recently proposed by the International Committee on Taxonomy of Viruses (ICTV), based on proteins involved in virion morphogenesis and/or in viral genome replication (Koonin et al., 2020). To date, only viruses from the realms *Duplodnaviria* and *Varidnaviria*, both corresponding mostly to double-stranded (ds) DNA viruses, infect hosts from the three domains of life (Koonin et al., 2020). These realms were previously recognized as lineages, based on the conservation of their major capsid proteins (MCPs). *Duplodnaviria* and *Varidnaviria* were known as the HK97 and the PRD1-adenovirus lineages, respectively (Bamford, 2003; Baker et al., 2005; Abrescia et al., 2012). The *Duplodnaviria* mostly consists of archaeal and bacterial viruses, whereas *Varidnaviria* are well represented in the virosphere associated with all three domains. This realm is thus an ideal subject to study the evolution of viruses in the context of the universal tree of life.

The *Varidnaviria* encompasses many very diverse families (hence its name, Various DNA viruses) (Table 1) (Koonin et al., 2019, 2020). They are all double-stranded DNA viruses, except the FLiP single-stranded DNA viruses (Laanto et al., 2017). Notably, the sizes of their virions vary from very small to the most gigantic ones among viruses. In the new ICTV taxonomy, *Varidnaviria* are divided into two kingdoms: *Bamfordvirae*, characterized by a single MCP with a double jelly roll (DJR) fold and *Helvetiavirae* characterized by two MCPs, each with a single jelly roll fold (SJR) (Koonin et al., 2020) (Table 1).

**TABLE 1:** Viruses of the realm *Varidnaviria*.

	MCP	pATPase	Domain	Kingdom	Phylum	Class	Order
*Poxviridae*	DJR	FtsK/HerA family	Eukarya	*Bamfordvirae*	*Nucleocytoviricota*	*Pokkesviricetes*	*Chitovirales*
*Asfarviridae*	DJR	FtsK/HerA family	Eukarya	*Bamfordvirae*	*Nucleocytoviricota*	*Pokkesviricetes*	*Asfuvirales*
*Phycodnaviridae*	DJR	FtsK/HerA family	Eukarya	*Bamfordvirae*	*Nucleocytoviricota*	*Megaviricetes*	*Algavirales*
*Mollivirus*	DJR	FtsK/HerA family	Eukarya	*Bamfordvirae*	*Nucleocytoviricota*	*Megaviricetes*	*Algavirales*
*Mimiviridae*	DJR	FtsK/HerA family	Eukarya	*Bamfordvirae*	*Nucleocytoviricota*	*Megaviricetes*	*Imitervirales*
*Ascoviridae*	DJR	FtsK/HerA family	Eukarya	*Bamfordvirae*	*Nucleocytoviricota*	*Megaviricetes*	*Pimascovirales*
*Iridoviridae*	DJR	FtsK/HerA family	Eukarya	*Bamfordvirae*	*Nucleocytoviricota*	*Megaviricetes*	*Pimascovirales*
*Marseilleviridae*	DJR	FtsK/HerA family	Eukarya	*Bamfordvirae*	*Nucleocytoviricota*	*Megaviricetes*	*Pimascovirales*
Polintoviruses	DJR	FtsK/HerA family	Eukarya	*Bamfordvirae*	*Preplasmiviricota*	/	/
*Lavidaviridae*	DJR	FtsK/HerA family	Eukarya	*Bamfordvirae*	*Preplasmiviricota*	*Maveriviricetes*	*Priklausovirales*
*Adenoviridae*	DJR	ABC family	Eukarya	*Bamfordvirae*	*Preplasmiviricota*	*Tectiliviricetes*	*Rowavirales*
STIV (*Turriviridae*)	DJR	FtsK/HerA family	Archaea/Bacteria	*Bamfordvirae*	*Preplasmiviricota*	*Tectiliviricetes*	*Belfryvirales*
Bam35	DJR	FtsK/HerA family	Bacteria	/	/	/	/
PRD1 *(Tectiviridae)*	DJR	FtsK/HerA family	Bacteria	*Bamfordvirae*	*Preplasmiviricota*	*Tectiliviricetes*	*Kalamavirales*
Toil	DJR	FtsK/HerA family	Bacteria	/	/	/	/
PM2 (*Corticoviridae*)	DJR	FtsK/HerA family	Bacteria	*Bamfordvirae*	*Preplasmiviricota*	*Tectiliviricetes*	*Vinavirales*
FLiP	DJR	/	Bacteria	/	/	/	/
Odin	DJR	/	Bacteria*	/	/	/	/
*Sphaerolipoviridae*	SJR	FtsK/HerA family	Bacteria/Archaea	*Helvetiavirae*	*Dividoviricota*	*Laserviricetes*	*Halopanivirales*

This table summarizes the shared genes between different families of this lineage. DJR, double jelly roll; SJR: single jelly roll; NCLDV, Nucleo-Cytoplasmic Large DNA Viruses; STIV, Sulfolobus turreted icosahedral virus; FLiP, Flavobacterium-infecting bacteriophage; MCP, major capsid protein; pATPase, packaging ATPase. *The group Odin includes only bacterial MAG, except for one MAG of Odinarchaeota.

The kingdom *Helvetiavirae* only includes viruses infecting archaea or bacteria. All known viruses of this kingdom are closely related and have been grouped into a single family, the *Sphaerolipoviridae* (Gil-Carton et al., 2015; Demina et al., 2017). It has been suggested that *Bamfordvirae* originated from *Helvetiavirae* by ancestral gene fusion of the SJR folds of their two MCPs (Krupovič and Bamford, 2008; Krupovic et al., 2020). In contrast to *Helvetiavirae*, the kingdom *Bamfordvirae* includes many families of viruses infecting members from the three domains. In the ICTV classification, *Bamfordvirae* have been divided into two phyla, *Nucleocytoviricota*, which includes all large to giant *Varidnaviria*, and *Preplasmiviricota*, which includes all small *Bamfordvirae* (Koonin et al., 2020).

The *Nucleocytoviricota*, formerly known as the Nucleo-Cytoplasmic Large DNA Viruses (NCLDVs), only infect eukaryotes, whereas *Preplasmiviricota* again includes viruses infecting members of the three domains. *Nucleocytoviricota* have been divided into two classes, *Megaviricetes* and *Pokkesviricetes* whereas *Preplasmiviricota* have been divided between *Maveriviricetes* and *Tectiliviricetes* (Table 1). It has been proposed that Polintoviruses belong to *Preplasmiviricota*. These elusive viruses are related to mobile elements called Polintons that carry genes encoding the MCP and packaging ATPases (pATPases) typical of *Varidnaviria* (Krupovic et al., 2014). *Maveriviricetes* include a single family, *Lavidaviridae* (also dubbed virophages) of viruses infecting the virocells of *Mimiviridae*, whereas *Tectiliviricetes* includes again several families of viruses infecting members of the three domains. The only *Tectiliviricetes* infecting eukaryotes correspond to *Adenoviridae*; all other *Tectiliviricetes* infecting prokaryotes, either archaea or bacteria. The best-known archaeal and bacterial *Tectiliviricetes* are small viruses, such as *Tectiviridae*, *Turriviridae*, and *Corticoviridae*, exemplified by the virus PRD1 infecting *Escherichia coli*, the virus STIV (Sulfolobus Turreted Icosahedral Virus) infecting *Sulfolobus*, and the virus PM2 infecting *Pseudoalteromonas*, respectively (San Martín and van Raaij, 2018; Yutin et al., 2018). The name *Tectiliviricetes* (Tectivirid-like and the suffix viricetes for class taxa) was designed from the best-studied virus of this class, the *Tectiviridae* PRD1. Most of these viruses are also known to integrate into bacterial or archaeal genomes (Yutin et al., 2018) or exist as free plasmids corresponding to defective viruses (Gaudin et al., 2014).

Besides the few representatives of *Turriviridae*, *Tectiviridae*, and *Corticoviridae* already known, Koonin and co-workers identified in metagenome-associated genomes (MAGs) many new lineages of archaeal and bacterial *Tectiliviricetes*. They proposed their classification into six groups based on sequence similarities networks of their MCPs and detection of signature proteins specific for each group (Table 1) (Yutin et al., 2018). The groups PM2, STIV and PRD1 could correspond to the orders *Vinavirales*, *Belfryvirales*, and *Kalamavirales* of the ICTV classification, respectively, whereas the Odin, Bam35/Toil and FLiP groups remained unclassified (Table 1). *Autolykiviridae*, a family of viruses abundant in marine microbial metagenomes (Kauffman et al., 2018) was included in the PM2 group by Koonin and colleagues (Yutin et al., 2018).

The group Odin was named after an integrated element present in the MAG of an Odinarchaeon, but all other members of this group were detected in bacterial MAGs. All other *Tectiliviricetes* infecting archaea were included in the group STIV, named from the archaeovirus STIV member. The STIV group itself was divided into two subgroups based on their MCP phylogeny, one including archaeoviruses and the other bacterioviruses (Yutin et al., 2018). The four other groups defined by Koonin and colleagues (PM2, PRD1, Bam35/Toil, and FLiP) include only bacterioviruses (Yutin et al., 2018) (Table 1).

Several hypotheses have been proposed regarding the origin and evolution of *Varidnaviria*. Several authors suggested that *Varidnaviria* predated the Last Universal Common Ancestor (LUCA) (Bamford et al., 2005) and that the transition from *Helvetiavirae* to *Bamfordvirae* took place before LUCA (Krupovic et al., 2020). Koonin, Krupovic, and colleagues even suggested that the diversification of bacterial and archaeal *Tectiliviricetes* predated LUCA (Krupovic et al., 2020). For these authors, *Varidnaviria* infecting eukaryotes originated later from a Tectivirus infecting the bacterium at the origin of mitochondria (Krupovic and Koonin, 2015). It is worth noting that, if this scenario is correct, the new ICTV ranking of *Varidnaviria* does not follow the rules of phylogenetic systematics, sensu Hennig (1965), since both *Helvetiavirae* and *Tectiliviricetes* are paraphyletic (e.g., the last common ancestor of *Helvetiavirae* was an ancestor of *Tectiliviricetes* and the last common ancestor of *Tectiliviricetes* was also an ancestor of all *Varidnaviria* infecting eukaryotes).

To evaluate the validity of the above scenario and eventually propose new ones, as well as to test the validity of the newly proposed ICTV classification, it is essential to determine the evolutionary history of *Varidnaviria* based on robust phylogenetic analyses. Several trees based on the structural comparison of MCPs from a set of *Bamfordvirae* representatives have been published (Ravantti et al., 2013, 2020). The evolutionary relationships among *Bamfordvirae* were also investigated using pairwise amino-acid sequence similarities networks (Sinclair et al., 2017; Yutin et al., 2018). Although such studies can provide interesting information for further analyses, they cannot completely replace sequence-based phylogenetic analyses in determining the actual evolutionary history of biological objects or organisms.

Until now, sequence-based phylogenetic analyses dealing with *Varidnaviria* have only focused on subgroups of *Bamfordvirae*. Most of them have specifically addressed the evolution of *Nucleocytoviricota* and the origin of giant viruses. They have shown that gigantism most likely originated several times independently during the evolution of *Nucleocytoviricota* (Guglielmini et al., 2019; Koonin and Yutin, 2019). The ICTV classification of this phylum is congruent with phylogenetic analyses of five marker genes conserved in most *Nucleocytoviricota* (Koonin and Yutin, 2019). However, in our recent phylogenetic analysis based on 8 marker genes (Guglielmini et al., 2019), we observed some discrepancies, in particular related to the position of *Asfarviridae* and related viruses. A global phylogeny of prokaryotic *Tectiliviricetes* based on their MCPs was also published (Kauffman et al., 2018). In this phylogeny, STIV-related archaeoviruses branched within *Tectiliviricetes* infecting bacteria, suggesting a virus transfer from Bacteria to Archaea. This is surprising since another recent phylogeny focusing on STIV-related archaeoviruses suggested that these viruses predated the last archaeal common ancestor (LACA) (Krupovic et al., 2019). The latter phylogeny was based on the concatenation of their MCP and their pATPases. Besides a common MCP, most members of *Varidnaviria* share indeed a homologous pATPase of the FtsK/HerA superfamily P-loop ATPases (Table 1) (Abrescia et al., 2012; Yutin et al., 2018). Concatenation of the MCP and pATPase proved also useful in rooting the phylogenetic tree of *Nucleocytoviricota* with the Polintoviruses as an outgroup (Guglielmini et al., 2019), questioning the possibility to extrapolate such an approach to the whole *Varidnaviria* realm.

Here, we have revisited the distribution and structural similarities of MCPs and pATPases among viruses of the realm *Varidnaviria* to identify the viruses that could be relevant to the evolutionary history of their morphogenesis module. We found that these two proteins can be used as phylogenetic markers for most members of the kingdom *Bamfordvirae*. We excluded *Adenoviridae*, Odin and FLiP groups from our analysis because they lack homologous pATPase and/or the sequences of their MCP are too divergent. The concatenation of the MCP and pATPase sequences produces a rather robust viral phylogeny that can be useful to infer the history of the *Bamfordvirae* morphogenesis module. *Helvetiavirae* were excluded from our concatenation because their MCPs were too divergent, but we could include them in our single pATPase tree. Our analyses validate some of the recent taxonomic proposals but challenge others, such as the ranking of *Sphaerolipoviridae* in a specific kingdom or the grouping of *Lavidaviridae*, Polintoviruses, and *Adenoviridae* within *Tectiliviricetes.* We confirm that *Tectiliviricetes* infecting archaea are closely related to those infecting bacteria, in contradiction with the topology of the universal cellular tree. We discuss the main hypothesis that has been previously proposed to describe the evolution of *Varidnaviria* in the light of our findings and explore alternative scenarios that could explain the discrepancy between the viral tree of *Bamfordvirae* and the universal cellular tree of life.

## Materials and Methods

### Selection of MCP/pATPase Sequences

Representative MCP/pATPase sequences from different groups of *Preplasmiviricota* were used as queries for PSI-BLAST (Altschul et al., 1997) searches against the GenBank non-redundant protein sequence database (nr). The query sequences are listed below:

**Table:** 

Group	Name	MCP	pATPase
*Lavidaviridae*	Sputnik virophage	YP_002122 381	YP_002122364
*Adenoviridae*	Frog adenovirus 1	NP_062443	NP_062434
STIV	Sulfolobus turreted icosahedral virus 1	YP_025022	YP_025021
Bam35	Bacillus phage Bam35c	NP_943764	NP_943760
*Tectiviridae*	Enterobacteria phage PRD1	NP_040692	NP_040689
Toil	Rhodococcus phage Toil	ARK07697	ARK07695
PM2	Pseudoaltero monas phage PM2	NP_049903	NP_049900
FLiP	Flavobacterium phage FLiP	ASQ41214	-
			

The MCP/pATPase sequences of the *Nucleocytoviricota* were retrieved from a previous study that we conducted (Guglielmini et al., 2019). The Polintoviruses sequences were gathered from the Repbase collection (Jurka et al., 2005)^1^ : Polinto-2_NV, Polinto-1_DY, Polinto-1_TC, Polinto-1_SP, Polinto-2_SP, Polinto-2_DR, and Polinto-1_DR. Finally, the sequences from the SJR group were recovered based on previously identified sequences (Demina et al., 2017).

Putative MCP/pATPase sequences were aligned with the query sequences for the examination of the conserved structural elements using MAFFT (Katoh and Standley, 2013). Prediction of the secondary structure was performed using Phyre2 (Kelley et al., 2015) and the predicted protein structures were visualized using UCSF Chimera (Pettersen et al., 2004). The sequences used in this study are shown in the Supplementary File. After removing sequences with no significant matches or low confidence levels, we obtained two different datasets of 145 and 128 sequences for the MCP and pATPase respectively.

### Network Analysis

After performing the structural protein prediction analysis, all-against-all blastp analyses were performed on the refined pATPase datasets. The all-against-all blastP results were grouped using the SiLiX (for *SIngle LInkage Clustering of Sequences*) package v1.2.8^2^ (Miele et al., 2011). This approach for the clustering of homologous sequences is based on single transitive links with alignment coverage constraints. The pATPase sequences were clustered independently by similarity using SiLiX with the expect threshold of 0.001 as previously used for MCP analysis (Yutin et al., 2018). The clustering results were analyzed and visualized using the igraph package of the R programming language^3^.

### Sequence Alignment

The alignments of the MCP sequences were performed using MAFFT v7.392 with the E-INS-i algorithm (Katoh and Standley, 2013), which can align sequences with several conserved motifs embedded in long unalignable regions, whereas pATPase sequences were aligned using MAFFT with the L-INS-i algorithm (Katoh and Standley, 2013), which can align a set of sequences containing sequences flanking around one alignable domain. Positions containing more than 30% of gaps were trimmed using goalign v0.2.8^4^.

### Phylogenetic Analysis

Single protein and concatenated protein phylogenies were conducted within the maximum likelihood (ML) framework using IQ-TREE v1.6.3 (Nguyen et al., 2015). We first performed a model test with the Bayesian Information Criterion (BIC) by including protein mixture models (Kalyaanamoorthy et al., 2017). For mixture model analyses, we used the PMSF models (Wang et al., 2018). Bootstrap trees with 1,000 replicates were generated using IQ-TREE with the same parameters as the best-known likelihood tree search. Non-parametric classical bootstrap values, as well as transfer bootstrap expectation (TBE) values (Lemoine et al., 2018) were computed using the software gotree v0.3.0^5^.

### Visualization

The phylogenetic trees were visualized with FigTree v1.4.3^6^ and iTOL (Letunic and Bork, 2007).

## Results

### Identification of *Varidnaviria* MCPs and pATPases Suitable for Phylogenetic Analysis

We retrieved MCP and pATPase sequences using PSI-BLAST searches against the NCBI non-redundant protein sequence database (nr) and added sequences recovered from proviruses (section “Materials and Methods”). We could identify both MCP and pATPase for most *Varidnaviria* (Table 1) with some exceptions. In particular, we could not detect putative pATPase in members of the Odin and FLiP groups, as was previously observed (Yutin et al., 2018). To validate the identified MCP and pATPase sequences, we generated protein models for all selected sequences and compared these predicted structures to the PDB database using Phyre2 (Supplementary Figure 1 and Supplementary Data File 1; Kelley et al., 2015). The *Varidnaviria* MCPs associated with groups and families previously described indeed matched their corresponding structures in the public databases, except for the putative MCPs identified from the Odin group, which was thus excluded from further analysis. The MCP from *Adenoviridae* was unique in exhibiting several additional structural elements (Supplementary Figure 1). We also confirmed that MCPs with SJR fold from *Sphaerolipoviridae* (*Helvetiavirae*) were very divergent from those of *Bamfordvirae.*

All *Varidnaviria* pATPases, except those of *Adenoviridae*, share similar predicted structures (Supplementary Figure 2 and Supplementary Data File 1) and clustered together in an amino acid sequence similarity network (Supplementary Figure 3). Previous observations based on amino-acid signatures and secondary structure predictions have indeed concluded that *Adenoviridae* pATPases were not specifically related to other *Varidnaviria* pATPases but to ATPases of the ABC superfamily (Supplementary Data File 1), indicating an exchange of pATPases during the evolution of *Adenoviridae* (Burroughs et al., 2007). Surprisingly, unlike the situation with MCP, the pATPases of *Sphaerolipoviridae* were structurally similar to those of *Bamfordvirae* (Supplementary Figure 2) and clustered together with *Tectiliviricetes* in our amino acid sequence similarity network (Supplementary Figure 3). Another peculiarity of the pATPase network was that *Lavidaviridae* exhibited more connections with *Tectiliviricetes* than with Polintoviruses and *Nucleocytoviricota*. Our results thus suggested that, besides the MCP, the pATPase gene could be an interesting marker for delineating the phylogeny of most *Varidnaviria* and could provide interesting information on the origin and evolution of *Sphaerolipoviridae.*

### Comparison of Single Trees Suggests Congruent Evolution of the MCPs and pATPases

**FIGURE 1:**
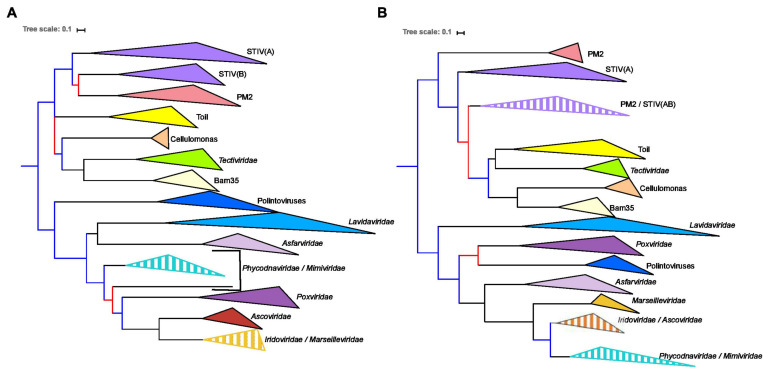
Single-protein trees of the two hallmark proteins of the viruses from the *Varidnaviria* realm, excluding *Adenoviridae, Sphaerolipoviridae*. Phylogenetic trees of **(A)** the major capsid protein (MCP) and **(B)** packaging ATPase (pATPase). The root of the phylogenetic tree was between the prokaryotic and eukaryotic members. The scale-bar indicates the average number of substitutions per site. The best-fit model for the MCP tree was LG + F + R4, which was chosen according to Bayesian Information Criterion (BIC) and the alignment has 103 sequences with 237 positions. The best-fit model for the pATPase tree was LG + R6, which was chosen according to BIC and the alignment has 103 sequences with 171 positions. More detailed versions of the trees are shown in Supplementary Figures 4, 5. Branches in black indicate both classical bootstrap and transfer bootstrap expectation (TBE) support values using 1,000 replicates are above 70%. Branches in blue indicate only one of the two support values is above 70% whereas branches in red indicate both support values are below 70%.

**FIGURE 2:**
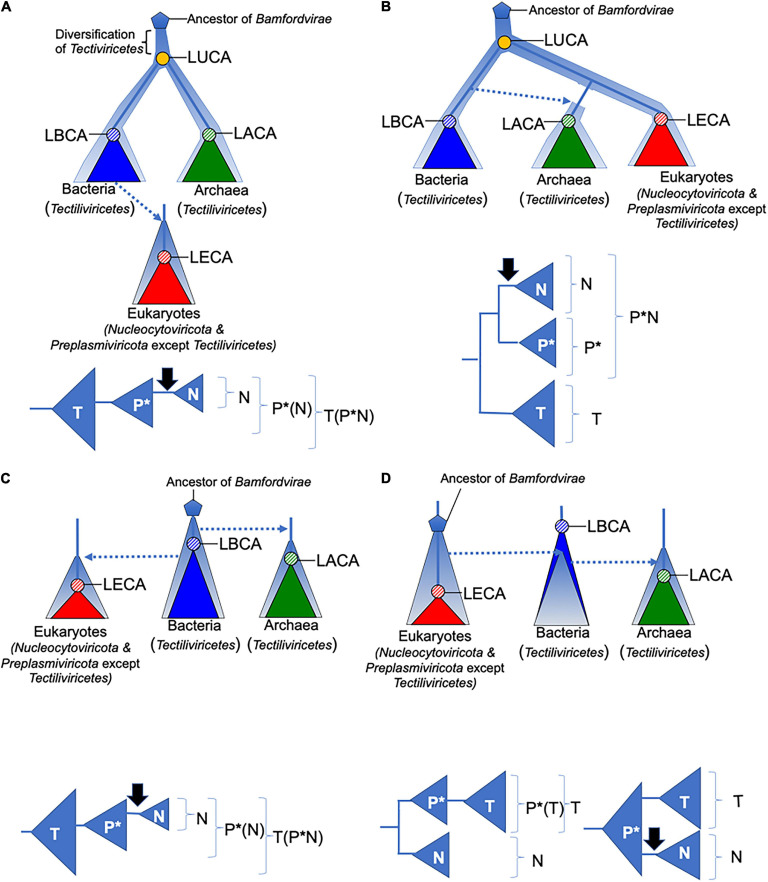
Four scenarios for the evolution of *Bamfordvirae*. The diversification and evolution of viruses are depicted with pale blue thick lines or triangles. Lower panels depict groups that are monophyletic (in brackets) among *Tectiliviricetes* (T), *Preplasmiviricota* except *Tectiliviricetes* (P*), and *Nucleocytoviricota* (N) in the above scenarios. The black arrows indicate the introduction of DNA replication proteins related to those of *Duplodnaviria* in the ancestral lineage of *Nucleocytoviricota*. Dotted arrows indicate transfer of viruses between different cellular domains. In all scenarios, we assumed that *Bamfordvirae* were present in the last archaeal common ancestor (LACA). In panels **(A,B)**, the origin of *Bamfordvirae* (blue pentagon) predated the last universal common ancestor (LUCA). In panel **(A)**, *Tectiliviricetes* emerged first and different lineages were selected and co-evolved with the proto-bacterial and proto-archaeal lineages. In panel **(B)**, the ancestral *Varidnaviria* thriving at the time of LUCA have now disappeared, *Tectiliviricetes* emerged in the bacterial branch and were later transferred to Archaea. The ancestors of eukaryotic *Bamfordvirae* were lost in Archaea. In panels **(C,D)**, *Bamfordvirae* originated after LUCA, either in proto-bacteria **(C)** or proto-eukaryotes **(D)**. They appeared later in the two other domains by virus transfer. LUCA, last universal common ancestor; LACA, last archaeal common ancestor; LBCA, last bacterial common ancestor; LECA, last eukaryal common ancestor.

To facilitate the comparison of the MCP and pATPase trees, we removed the taxa that were not present in both datasets, *Helvetiavirae*, FLiP, and Odin, as well as *Adenoviridae* whose MCP and pATPase could not be aligned with those of other *Varidnaviria* (Table 1). Phylogenetic analyses were first performed separately on the two proteins within the ML framework (section “Materials and Methods”). We thus obtained the first sequence-based phylogenies covering most groups of *Bamfordvirae* (Figure 1 and Supplementary Figures 4, 5). The MCP and pATPase trees exhibited noticeable congruence confirming that they belong to the same module (Iranzo et al., 2016b) (Figure 1). Notably, *Bamfordvirae* infecting prokaryotes (*Tectiliviricetes*) and those infecting eukaryotes formed two distinct clusters with good TBE support values. The only large bipartition in common between the two single-protein trees while maintaining most known large monophyletic groups corresponded to the separation between *Tectiliviricetes* and eukaryotes-infecting *Bamfordvirae*. We thus decided to root the trees between these two clusters (Figure 1) although there are other possibilities (see section “Discussion” and Figure 2).

We recovered in both trees the monophyly of most previously defined groups and/or families of eukaryoviruses. Noticeably, the *Nucleocytoviricota* phylum was not monophyletic because of the variable positions of *Asfarviridae* and *Poxviridae*. We also recovered the monophyly of the previously defined groups of *Tectiliviricetes*, except for the STIV group that was paraphyletic in both trees and the PM2 group that was paraphyletic in the pATPase tree. STIV infecting archaea and STIV infecting bacteria were monophyletic in the MCP tree but the latter were sister group to the PM2 group. In the pATPase tree, some STIV infecting archaea were mixed with the PM2 group. Noticeably, the archaeoviruses STIV branched within bacterioviruses in both cases, as previously observed (Kauffman et al., 2018). Other similarities between the MCP and pATPase trees were the grouping of the bacterioviruses STIV with the PM2 group and the proximity of the Cellulomonas, Bam35, *Tectiviridae* in both trees (the three of them forming a single clade together with the Toil group in the pATPase tree).

The small differences observed between the MCP and the pATPase trees could be due to conflicting phylogenetic signals due to lateral gene transfer and/or to the low resolution in some part of these trees. The rather good congruence between the two trees thus suggested that concatenation of the MCP and pATPase sequences could be used to obtain a more reliable phylogeny of the *Bamfordvirae* virion morphogenesis module.

### Concatenation of the MCP and the pATPase Produces a Robust Tree of *Bamfordvirae*

**FIGURE 3:**
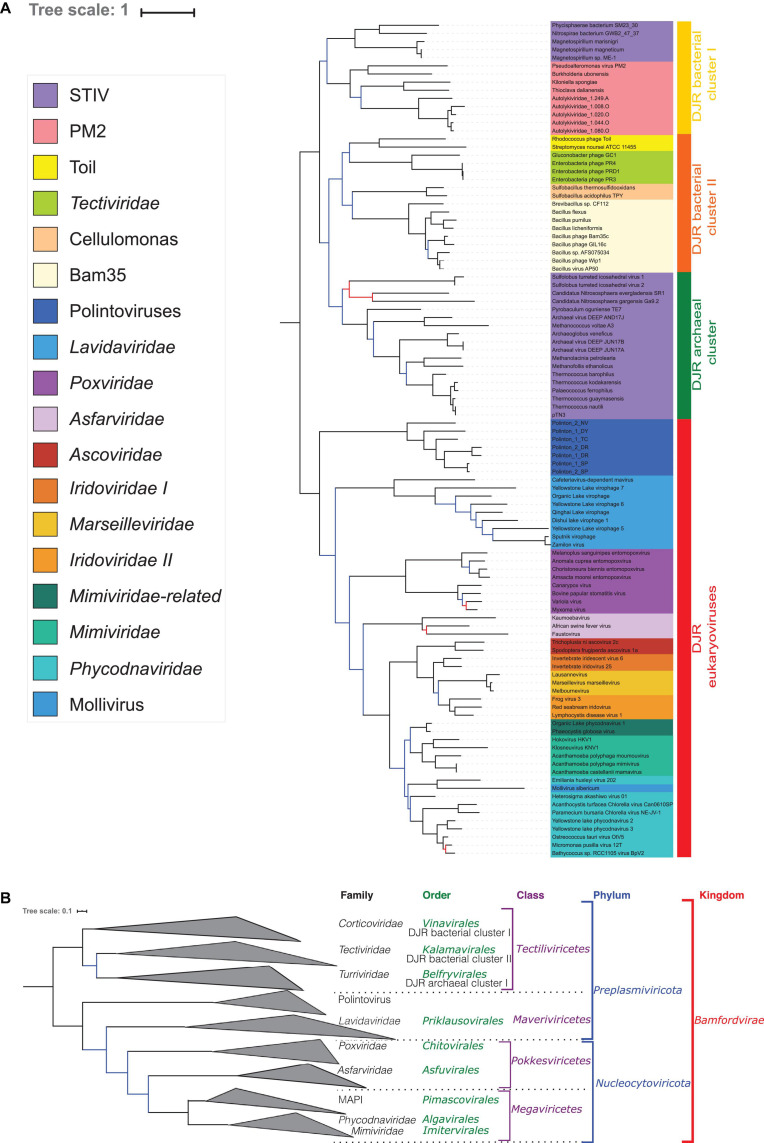
Phylogenetic tree of the concatenated MCP and pATPase genes. **(A)** Phylogenetic tree annotated with the corresponding group. The alignment has 103 sequences with 408 positions. Phylogenetic tree was rooted between the prokaryotic and eukaryotic members. The scale-bar indicates the average number of substitutions per site. The best-fit model was LG + F + R6, which was chosen according to Bayesian Information Criterion (BIC). Branches in black indicate both classical bootstrap and transfer bootstrap expectation (TBE) support using 1,000 replicates values are above 70%. Branches in blue indicate only one of the two support values is above 70% whereas branches in red indicate both support values are below 70%. **(B)** Phylogenetic tree annotated with the ICTV taxonomy.

In the concatenated tree, the number of well-supported branches increased (Figure 3A and Supplementary Figure 6). In particular, we recovered both strong bootstraps (0.95) and TBE support (0.99) for the bipartition between *Bamfordvirae* infecting eukaryotes and those infecting prokaryotes (Figure 3A and Supplementary Figure 6) although TBE supports for the grouping of *Tectiliviricetes* with Polintons (0.91) or with Polintons and *Lavidaviridae* (0.88) were also rather high. We recovered most clades suggested by the ICTV classification, except for the *Pokkesviricetes* (Figure 3B). The ICTV classification would have suggested rooting the tree between *Lavidaviridae* and *Poxviridae* (Figure 3B) (see the discussion for the different possible positions of the root). In contrast to the results obtained with the single-protein trees, *Nucleocytoviricota*, including *Poxviridae*, were monophyletic in the concatenated tree. When rooting between the eukaryoviruses and bacterioviruses, the Polintoviruses were basal to all eukaryotic groups. The *Poxviridae* were the first branching family of *Nucleocytoviricota*, followed by the *Asfarviridae*. Notably, we recovered the two major groups of *Nucleocytoviricota* that we previously identified based on 8 core genes (Guglielmini et al., 2019), the MAPI (*Marseilleviridae*, *Ascoviridae*, Pitho-like viruses, and *Iridoviridae*), which corresponds to the order “*Pimascovirales”* and the PAM [*Phycodnaviridae* (*Algavirales*), *Asfarviridae* (*Pokkesviricetes*), and Megavirales (*Imitervirales*)] except that the *Asfarviridae* were a sister group to these two superclades instead to be part of the PAM.

Interestingly, whereas the STIV group remained paraphyletic in the concatenated trees, the STIV viruses infecting archaea formed a monophyletic group (hereinafter called archaeal DJR cluster) with a good TBE support (0.88) (Figure 3 and Supplementary Figure 6), as in the MCP tree (Figure 1A). The *Tectiliviricetes* infecting bacteria were divided into two clades. The first one (hereinafter called DJR bacterial cluster I) contained the PM2 group, including *Autolykiviridae*, and bacterial members of the STIV group. The TBE support for this group was rather weak (0.70) but still significant. The second clade, (hereinafter called DJR bacterial cluster II) was strongly supported (TBE = 0.90) contained the PRD1, Cellulomonas and the Bam35/Toil groups. The DJR bacterial cluster I emerged at the base of the *Tectiliviricetes*, whereas the DJR bacterial cluster II was the sister group to the archaeal DJR cluster with low but significant support.

**FIGURE 4:**
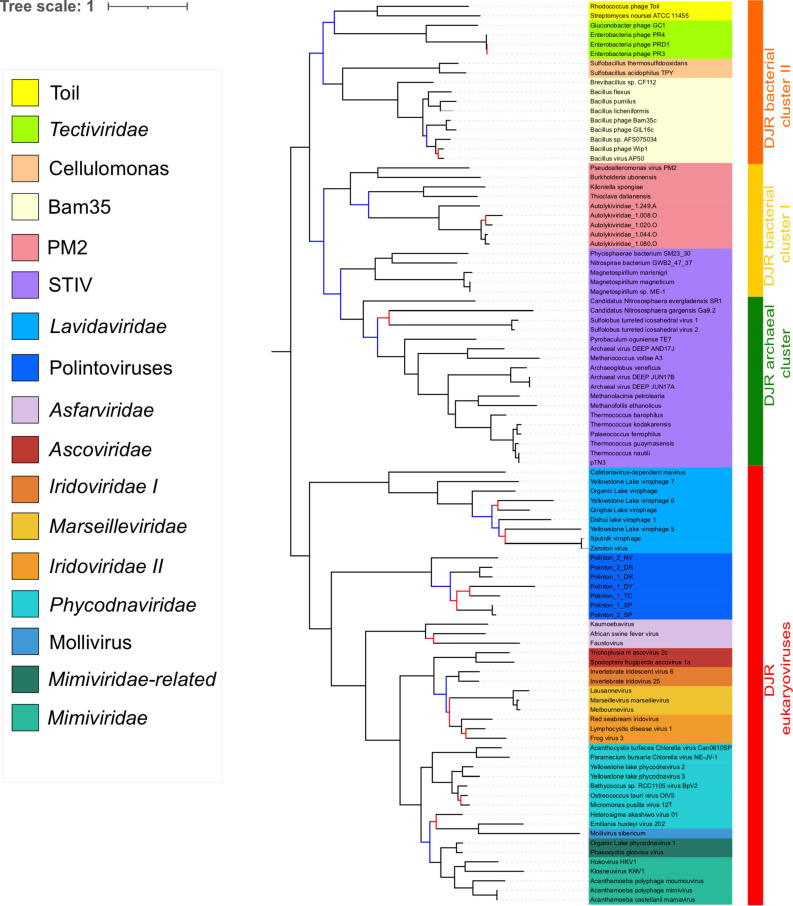
Phylogenetic tree of the concatenated major capsid protein and packaging ATPase genes, excluding the *Poxviridae*. The alignment has 95 sequences with 434 positions. Phylogenetic tree was rooted between the prokaryotic and eukaryotic members. The scale-bar indicates the average number of substitutions per site. The best-fit model was LG + F + R5, which was chosen according to Bayesian Information Criterion (BIC). Branches in black indicate both classical bootstrap and transfer bootstrap expectation (TBE) support values using 1,000 replicates are above 70%. Branches in blue indicate only one of the two support values is above 70% whereas branches in red indicate both support values are below 70%.

We previously noticed that *Poxviridae* have long branches and variable positions in single-gene trees of *Nucleocytoviricota* proteins (Guglielmini et al., 2019). In particular, they tended to attract the long branches of the *Asfarviridae* in our previous analyses, forming a clade corresponding to the recently proposed class *Pokkesviricetes*. When we removed *Poxviridae* from our dataset to prevent possible long-branch attraction, we obtained a tree in which *Nucleocytoviricota* are no more sister group to the *Lavidaviridae* (Figure 4 and Supplementary Figure 7), but Polintoviruses, with good support. In that case, *Lavidaviridae* branched between *Tectiliviricetes* and all other *Varidnaviria*, as in the pATPase tree. This position could also explain their weak clustering with *Tectiliviricetes* in the pATPase amino-acid similarity network (Supplementary Figure 3). The monophyly of archaeal *Tectiliviricetes*, previously observed with the complete MCP tree, was even more strongly supported (TBE = 0.97) in the concatenated trees without *Poxviridae* (Figure 4 and Supplementary Figure 7). In addition, we obtained with strong TBE support the monophyly of the entire STIV group, STIV archaeoviruses branching as sister group to STIV bacterioviruses. In this tree, the DJR archaeal cluster was thus included in the DJR bacterial cluster I which became paraphyletic, whereas the DJR bacterial cluster II emerged with strong TBE support at sister clade to a clade grouping the archaeal *Tectiliviricetes* and bacterial *Tectiliviricetes* of the DJR bacterial cluster (Figure 4 and Supplementary Figure 7).

### *Sphaerolipoviridae* (*Helvetiavirae*) Branch With *Tectiliviricetes* (*Bamfordvirae*) in the pATPase Tree

**FIGURE 5:**
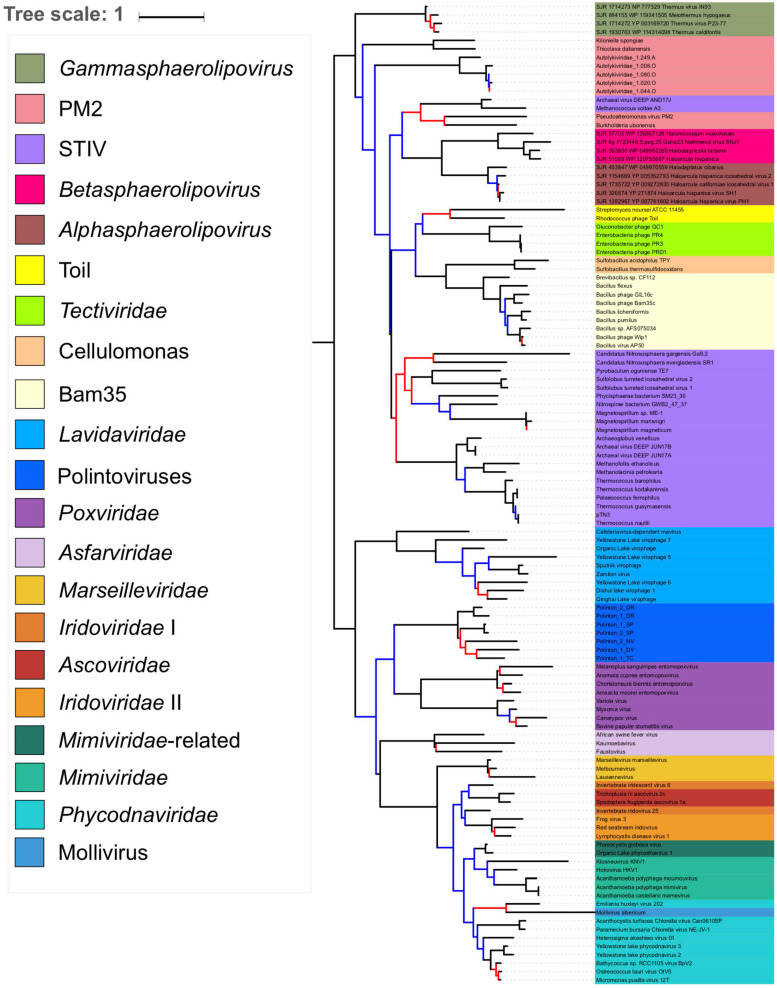
Phylogenetic tree of the packaging ATPase gene of the *Varidnaviria* realm, excluding *Adenoviridae.* The alignment has 116 sequences with 151 positions. The root of the phylogenetic tree was between the prokaryotic and eukaryotic members. The scale-bar indicates the average number of substitutions per site. The best-fit model was LG + R6, which was chosen according to Bayesian Information Criterion (BIC). Branches in black indicate both classical bootstrap and transfer bootstrap expectation (TBE) support values using 1,000 replicates are above 70%. Branches in blue indicate only one of the two support values is above 70% whereas branches in red indicate both support values are below 70%.

As suggested by the pATPase amino-acid similarity network (Supplementary Figure 3) we could add the *Sphaerolipoviridae* pATPases sequences to our pATPase alignment. We thus obtained a pATPase tree in which *Sphaerolipoviridae* were grouped with *Tectiliviricetes*, as in our network analysis (Figure 5 and Supplementary Figure 8). The relative position of the different groups of *Tectiliviricetes* in the pATPase tree remained somewhat similar to the same tree before the inclusion of *Sphaerolipoviridae* (Figure 1) except clear differences involving the STIV and PM2, probably due to a complex evolutionary pattern or a lack of resolution. *Sphaerolipoviridae* did not form a single monophyletic group basal to both eukaryoviruses and prokaryoviruses, as would have been expected in the SJR to DJR scenario, but two strongly supported monophyletic groups. Gamma Sphaerolipoviruses, which infect thermophilic bacteria, branched at the base of *Tectiliviricetes*, whereas Alpha and Beta Sphaerolipoviruses, which infect halophilic archaea branched deeper among *Tectiliviricetes*, forming a clade branching with two PM2 and two archaeal STIV sequences. This suggests two different origins for archaeal and bacterial SJR pATPases.

## Discussion

It has long been thought that it was not possible to build a valid sequence-based phylogeny of viruses infecting members of the three cellular domains. Here, we have obtained a rather well resolved and informative phylogeny for the realm *Varidnaviria*, based on the concatenation of their MCPs and pATPases. A similar strategy has been recently adopted to produce a global evolutionary history of the realm *Riboviria* (RNA viruses) based on the phylogeny of a single protein, their RNA-dependent RNA polymerases (Wolf et al., 2018). In the case of *Varidnaviria*, proteins involved in DNA replication cannot be used because different groups use non-homologous DNA replication proteins. However, a tree based on MCPs and pATPases might better correspond to what we expect for a “viral tree” if the virion and its mode of formation are considered to be the hallmark of the virus (Bamford, 2003; Krupovic and Bamford, 2010; Forterre et al., 2014; Krupovic et al., 2020). We looked for the possibility to use additional proteins in virion morphogenesis, such as the minor capsid protein (mCP) and cysteine protease (PRO) to increase the robustness of our tree. However, mCPs and PROs are not well conserved in *Varidnaviria*. For instance, PROs have not been identified among *Tectiliviricetes*. The relatively small gene size of mCP and PRO also limits their usefulness in inferring phylogeny.

We could not include *Adenoviridae* and *Sphaerolipoviridae* in our concatenation because they did not encode the canonical MCP and/or pATPase. The MCP of *Adenoviridae* is too divergent from those of other *Varidnaviria*, whereas their pATPases belongs to another superfamily of P-loop ATPase (Burroughs et al., 2007). *Adenoviridae*, which have rather small genomes, have been tentatively included by the ICTV in the phylum *Preplasmiviricota* and the class *Tectiliviricetes* (Koonin et al., 2020) (Table 1). However, *Adenoviridae* exhibit more connection with *Nucleocytoviricota* than with *Tectiliviricetes* in a sequence similarity network (Sinclair et al., 2017) and branch far from *Tectiliviricetes* in a recent MCP structural tree (Ravantti et al., 2020), suggesting that classification of *Adenoviridae* within *Tectiliviricetes* could be premature.

In agreement with the classification of *Sphaerolipoviridae* as a distinct kingdom, *Helvetiavirae*, their MCP cannot be confidently aligned with those of *Bamfordvirae*. However, surprisingly, their pATPases could be aligned with those of *Bamfordvirae* and branched with *Tectiliviricetes* in the pATPase tree. Moreover, the pATPases of Alpha and Beta Sphaerolipoviruses and those of Gamma Sphaerolipoviruses branched at different positions (Figure 5). It was proposed that the single MCP with two jelly roll folds of *Bamfordvirae* originated by gene fusion of the two MCP of *Sphaerolipoviridae* (Krupovič and Bamford, 2008; Krupovic et al., 2020). However, our results suggest an alternative hypothesis, i.e., that the *Sphaerolipoviridae* SJR MCP originated twice from two distinct Tectivirus-like viruses by the deletion of one of the two jelly roll folds, followed by gene duplication. In that case, the MCP sequences of *Sphaerolipoviridae* might have rapidly diverged from those of *Bamfordvirae* following their structural rearrangements. An argument favoring the scenario from DJR to SJR might be the narrow distribution of *Sphaerolipoviridae*. In Archaea, *Sphaerolipoviridae* are only known infecting Haloarchaea, whereas in Bacteria, they only infect *Thermus* species, suggesting a “recent” emergence of these viruses. In contrast, the SJR to DJR scenario implies that *Sphaerolipoviridae* are very ancient, possibly predating LUCA (Krupovic et al., 2020). In that case, one would have expected a large distribution of these viruses in the three domains. Future exploration of the *Varidnaviria* diversity will possibly help to determine the correct scenario. In the meantime, it seems premature to consider the single family *Sphaerolipoviridae* as the prototype for a new kingdom.

Since we could not include *Sphaerolipoviridae* (*Helvetiavirae*) in our concatenated tree, this tree is formally a tree of *Bamfordvirae*. Noticeably, we recovered the monophyly of most families/groups previously defined on different criteria (Yutin et al., 2018). In particular, the internal phylogeny of the *Nucleocytoviricota*, with the monophyly of the order *Pimascovirales* (formerly the MAPI cluster) is very similar to the one that we previously obtained with eight-core genes of *Nucleocytoviricota*, the only difference being the position of *Asfarviridae* (Guglielmini et al., 2019). *Poxviridae* and *Asfarviridae* did not form a monophyletic group in our concatenated MCP/pATPase trees (Figures 3, 4), in contradiction with findings of other studies, which recovered the clade with *Poxviridae* and *Asfarviridae* (Fischer et al., 2010; Hingamp et al., 2013), and their ICTV classification into the proposed class *Pokkesviricetes*. The *Asfarviridae* branch between Polintoviruses and *Nucleocytoviricota* in our *Bamfordvirae* tree without *Poxviridae* (Figure 4), whereas they emerged within Megaviricetes in our previous concatenation of the MCP and pATPase, which was limited to *Nucleocytoviricota* (except *Poxviridae*) and Polintoviruses (Guglielmini et al., 2019). The grouping of *Asfarviridae* with Megaviricetes was also observed in the MCP structural tree of Ravantti et al. (2020). We did not recover this grouping here and this is possibly due to long-branch attraction of *Asfarviridae* by the out-group sequences.

Our analysis supports the grouping of archaeal and bacterial *Bamfordvirae* in the same rank (*Tectiliviricetes*). There was a robust cluster (DJR cluster II) including the PRD1 (*Tectiviridae*), Bam35, Cellulomonas and Toil groups, which could correspond to the proposed ICTV order *Kalamavirales*. We also obtained the monophyly of archaeal STIV, which could correspond to the proposed ICTV order *Belfryvirales* and a robust cluster grouping members of the PM2 group and *Autolykiviridae*, as already suggested by Koonin and colleagues (Yutin et al., 2018). PM2 and relatives have been classified by the ICTV in the order *Vinavirales*. The position of bacterial STIV remains uncertain, they are sister group to the PM2 group in our concatenated tree with *Poxviridae* (Figure 3), suggesting classifying them in the order *Vinavirales* or a new order; in contrast, they form a monophyletic group with archaeal STIV in the concatenated tree without *Poxviridae* (Figure 4), suggesting to classify them in the order *Belfryvirales*.

*Lavidaviridae* (virophages) and Polintoviruses have been grouped with *Tectiliviricetes* in the same phylum, *Preplasmiviricota* based on a gene network analysis that has defined a Polinton-like module also including cytoplasmic and mitochondrial plasmids (Iranzo et al., 2016b) (hence the name *Preplasmiviricota*, *meaning* precursor of certain plasmids). This phylum was not recovered in our single-protein phylogenies, since either *Lavidaviridae* or Polintoviruses branch with *Poxviridae* that belong to *Nucleocytoviricota.* Although these branching are probably due to long branch attraction between *Poxviridae* and these two groups, it could also reflect a closer relationship of these two groups for *Nucleocytoviricota* than for *Tectiliviricetes*, as previously observed in a sequence similarity network (Sinclair et al., 2017). However, the grouping of *Lavidaviridae* or Polintoviruses with *Tectiliviricetes* is neither specifically supported nor refuted in our concatenated phylogeny since *Lavidaviridae* and Polintoviruses branched between *Tectiliviricetes* and *Nucleocytoviricota*.

We have previously shown that *Nucleocytoviricota* have already diverged before the Last Eukaryotic Common Ancestor (LECA) (Guglielmini et al., 2019). The present study indicates that *Nucleocytoviricota*, *Lavidaviridae*, and Polintoviruses should have diverged even earlier and co-evolved for a long time with proto-eukaryotes. The early divergence of *Lavidaviridae* and *Nucleocytoviricota* is intriguing since all known modern *Lavidaviridae* (virophages) are parasites of *Imitervirales.* It suggests that the ancestors of modern *Lavidaviridae* used to infect proto-eukaryotes instead of giant viruses and were able to infect viruses present in their hosts subsequently. Integrated genomes of *Lavidaviridae* are abundant in some eukaryotes and used as tools to fight invading *Imitervirales* (Fischer and Hackl, 2016; Berjón-Otero et al., 2019). One can wonder if some *Lavidaviridae* are still able to infect eukaryotes in the absence of *Imitervirales* infection.

In Figure 2, we illustrate several of the possible scenarios for the evolution of *Bamfordvirae* (except *Adenoviridae*) and their implications for viral taxonomy, assuming that the structural module represents the vertical evolution of viruses. Koonin et al. (2006) proposed that *Tectiliviricetes* were already diversified at the time of LUCA and *Bamfordvirae* infecting eukaryotes evolved from a tectivirus that infected the bacterium at the origin of mitochondria (Figure 2A). They suggested that Polintoviruses originated first and became the ancestor of *Lavidaviridae* and *Nucleocytoviricota* (Krupovic and Koonin, 2015). Our MCP/pATPase concatenated tree does not support this specific version of their scenario since eukaryoviruses infecting *Bamfordvirae* did not branch within *Tectiviridae*. However, it is compatible with a rather similar scenario in which *Bamfordvirae* infecting eukaryotes evolved from an archaeal or a bacterial virus belonging to an extinct group of *Tectiliviricetes*. Notably, in such a scenario, the tree should be rooted within *Tectiliviricetes* and both *Tectiliviricetes* and *Preplasmiviricota* are paraphyletic (Figure 2A).

Our phylogenetic analysis produces a “viral tree of life” strikingly different from the cellular tree based on universal proteins in which either Archaea and eukaryotes are sister group or eukaryotes emerged within Archaea (Figure 2B) (Spang et al., 2015, 2018; Da Cunha et al., 2017, 2018), since *Bamfordvirae* infecting archaea and bacteria are grouped and separated from those infecting eukaryotes. In the scenario proposed by Koonin and colleagues, this contradiction is explained by the fact that modern *Tectiliviricetes* infecting archaea and bacteria have remained nearly identical to their ancestors 3–4 billion years ago, whereas the modern descendants of the tectivirus at the origin of *Bamfordvirae* infecting eukaryotes rapidly evolved into an immense variety of viral group, giving rise to *Lavidaviridae*, Polintoviruses and *Nucleocytoviricota* (Figure 2A).

The *Bamfordvirae* “viral tree of life” can be also explained in the framework of the classical Woese’s tree of life. For instance, one can imagine that the ancestral *Bamfordvirae* common to archaea and eukaryotes were lost in the proto-archaeal lineage and replaced by *Bamfordvirae* of the bacterial type via mobilome transfer (Figure 2B). This scenario cannot be excluded since such mobilome transfer has been previously proposed in the case of conjugative plasmids (Guglielmini et al., 2013). The transfer of several components of the bacterial mobilome to proto-archaea could explain why the mobilomes of archaea and bacteria are very similar to each other while being very different from the eukaryotic mobilome (Forterre, 2013). In such a scenario, the concatenated tree could be rooted between *Tectiliviricetes* and *Bamfordvirae* infecting eukaryotes. Noticeably, *Nucleocytoviricota* and a clade corresponding to *Preplasmiviricota* except *Tectiliviricetes* (P^∗^) form three monophyletic clades in that scenario (Figure 2B).

In the two scenarios previously discussed, we assumed that *Varidnaviria* were already present at the time of LUCA (Figures 2A,B). Another possibility is that *Varidnaviria* originated more recently (Figures 2C,D). In that case, considering the greater diversity of *Bamfordvirae* in Bacteria than in Archaea, it is tempting to imagine that these viruses originated in the bacterial lineage, suggesting a root within bacterial *Tectiliviricetes* (Figure 2C). If *Tectiliviricetes* were already present in the LACA as suggested by our analysis, this scenario again implies that both archaeal *Tectiliviricetes* and *Bamfordvirae* infecting eukaryotes originated from bacterial ones (Figure 2C). As in the case of the scenario of Figure 2A, *Tectiliviricetes* and *Preplasmiviricota* are both paraphyletic. Finally, an alternative version of a post-LUCA scenario is that *Bamfordvirae* originated in proto-eukaryotes and that some of them (related to Polintovirus/*Lavidaviridae*) were later on transferred to Bacteria, and finally from Bacteria to Archaea (Figure 2D). In that case, the MCP/ATPase tree could be rooted either between *Nucleocytoviricota* and *Preplasmiviricota* (including *Tectiliviricetes*) or within *Preplasmiviricota*, *Tectiliviricetes* forming a monophyletic group included in *Preplasmiviricota*. Although in contradiction with the current view suggesting that eukaryoviruses always originated from viruses infecting prokaryotes (Koonin et al., 2015), this hypothesis could explain why *Bamfordvirae* are so diverse and abundant in eukaryotes.

Interestingly, *Nucleocytoviricota* and *Preplasmiviricota* have strikingly different DNA replication proteins. In particular, *Nucleocytoviricota* share several of their major DNA replication proteins with head and tailed bacteriophages (*Caudovirales*) of the realm *Duplodnaviria* (Iranzo et al., 2016a). If *Nucleocytoviricota* originated from *Preplasmiviricota*, as suggested by Koonin and colleagues (Figures 2A,C), one should imagine that the DNA replication proteins encoded by the preplasmivirus at the origin of *Nucleocytoviricota* were replaced by the DNA replication proteins of *Caudovirales* infecting the same proto-eukaryotic hosts (black arrows on Figure 2). Such a replacement is also required in the scenario of Figures 2B,D (lower right panel). However, if the root of the *Bamfordvirae* tree is located between *Nucleocytoviricota* and *Preplasmiviricota* (Figure 2D, lower left panel), one can simply imagine that the divergence between these two phyla coincided with the association of an ancestral common virion morphogenesis module of the DJR type with two different types of replication modules, the replication modules that became associated with *Nucleocytoviricota* being a relative of the replication modules of some *Caudovirales*.

For some authors, the determination of viral phylogeny based on protein sequences comparison is a futile or at least risky exercise because they originated from different cell lines and that the core proteins characteristic of a modern viral lineage could be an artifact due to the random losses of proteins initially present their cellular ancestors (Claverie, 2020; Nasir et al., 2020). This is probably not the case for the core proteins of *Nucleocytoviricota* since we have shown that the concatenation and single phylogenies of their eight-core genes were mostly congruent (Guglielmini et al., 2019). In any case, our result indicates that it is possible to trace the origin of the *Varidnaviria* virion morphogenesis module to a common ancestor that was already a virus, i.e., an organism that used the production of virions as the mode of dissemination of its genome (Raoult and Forterre, 2008), the origin and nature of this ancestral varidnavirus remaining enigmatic.

## Conclusion

The presence of *Varidnaviria* in the three cellular domains raises challenging questions about their origin and evolution. Here, we have shown that phylogenies based on the concatenation of their MCP and pATPase can help to validate and/or question the viral classification and nomenclature of *Varidnaviria* recently proposed by the ICTV and can be used as a backbone to discuss current hypotheses about their evolution and propose new ones. In particular, we confirm the monophyly of *Tectiliviricetes* and *Nucleocytoviricota* and we identified a robust clade of *Tectiliviricetes* corresponding to the DJR cluster II. The tree presented here is not yet stable, as indicated by the fact that adding or removing some lineages impacts the relationships between some major clades. It will thus certainly be improved in the future with the discovery of new viral groups and the discovery of new members of the existing groups. Future identification and isolation of new viral families of *Varidnaviria*, especially in cellular lineages that have been poorly investigated until now, will thus be essential to possibly choose between the various scenarios for the history of this fascinating realm.

## Data Availability Statement

The datasets presented in this study can be found in online repositories. The names of the repository/repositories and accession number(s) can be found in the article/Supplementary Material.

## Author Contributions

AW and PF designed the study. AW, JG, and VD performed the bioinformatics experiments. AW, JG, MG, VD, and PF analyzed and interpreted the results. AW, MG, VD, and PF wrote the manuscript. All authors contributed to the article and approved the submitted version.

## Conflict of Interest

The authors declare that the research was conducted in the absence of any commercial or financial relationships that could be construed as a potential conflict of interest.

## Footnotes

1 http://www.girinst.org/Repbase_Update.html

2 http://lbbe.univ-lyon1.fr/SiLiX

3 https://igraph.org/

4 https://github.com/evolbioinfo/goalign

5 https://github.com/evolbioinfo/gotree

6 http://tree.bio.ed.ac.uk/software/figtree/
