# Supplementary material for: Phylogeny of the Varidnaviria Morphogenesis Module: Congruence and Incongruence With the Tree of Life and Viral Taxonomy
Source: Front Microbiol. 2021 Jul 16;12:704052. doi: 10.3389/fmicb.2021.704052 (PMC8328091; doi:10.3389/fmicb.2021.704052)
Supplement: Supplementary file 1 [file Data_Sheet_1.pdf]

Supplementary Material

1 Supplementary Data

Supplementary Data 1. Prediction of the secondary structure of the sequences using Phyre2

Additional data (sequences, alignment files and tree files) related to this article may be obtained here: (https://zenodo.org/record/4953926#.YMhUVxMzbOQ)

2 Supplementary Figures

Supplementary Figure 1. Major capsid proteins of the *Varidnaviria* realm.

Supplementary Figure 2. Packaging ATPases of the *Varidnaviria* realm.

Supplementary Figure 3. Sequence similarity networks of the packaging ATPase proteins from the *Varidnaviria* realm.

Supplementary Figure 4. Phylogenetic tree of the major capsid protein gene of the *Varidnaviria* realm, excluding *Adenoviridae, Sphaerolipoviridae*, Odin and FLiP members.

Supplementary Figure 5. Phylogenetic tree of the packaging ATPase gene of the double jelly-roll viruses from the *Varidnaviria* realm, excluding *Adenoviridae* and *Sphaerolipoviridae*.

Supplementary Figure 6. Phylogenetic tree of the concatenated MCP and pATPase genes.

Supplementary Figure 7. Phylogenetic tree of the concatenated major capsid protein and packaging ATPase genes, excluding the *Poxviridae*.

Supplementary Figure 8. Phylogenetic tree of the packaging ATPase gene of the *Varidnaviria* realm, excluding *Adenoviridae.*

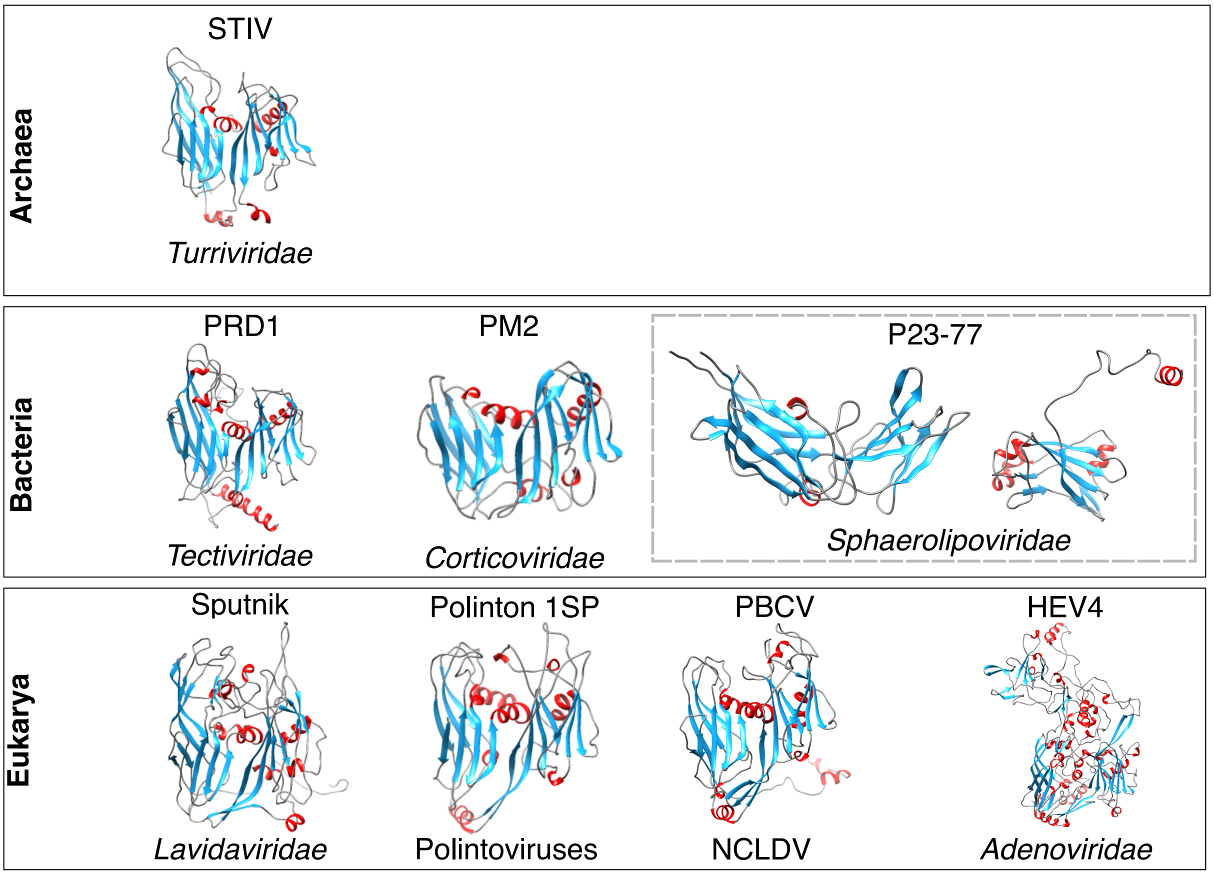

Supplementary Figure 1. Major capsid proteins of the *Varidnaviria* realm. Major capsid proteins (MCPs) with the double jelly-roll (DJR) fold and single jelly-roll (SJR) fold (boxed in dashed lines) are arranged horizontally according to the domain of life to which their hosts are classified. Virus names are provided above the structural models of various families of bacterial, archaeal and eukaryotic viruses. The structures are coloured according to the secondary structure elements: α-helices in red; β-strands in blue; and random coil in grey. The X-ray structure of the major capsid protein of Polinton 1 SP is not available and is represented with homology-based model. Sputnik, Sputnik virophage; Polinton 1 SP, Polinton 1 Strongylocentrotus purpuratus; PBCV, Paramecium bursaria Chlorella virus; NCLDV, nucleo-cytoplasmic large DNA viruses; HAE4, Human adenovirus E4; PRD1, Enterobacteria phage PRD1; PM2, Pseudoalteromonas phage PM2; STIV, Sulfolobus turreted icosahedral virus. P23-77, Thermus virus P23-77. RCSB Protein Data Bank (PDB) accession numbers for the major capsid protein structures: Sputnik, PDB entry 3J26; PBCV, PDB entry 5TIP; HAE4, PDB entry 2BVI; PRD1, PDB entry 1HB5; PM2, PDB entry 2W0C; STIV, PDB entry 2BBD; P23-77, PDB entry 3ZMN (left) and 3ZN4 (right).

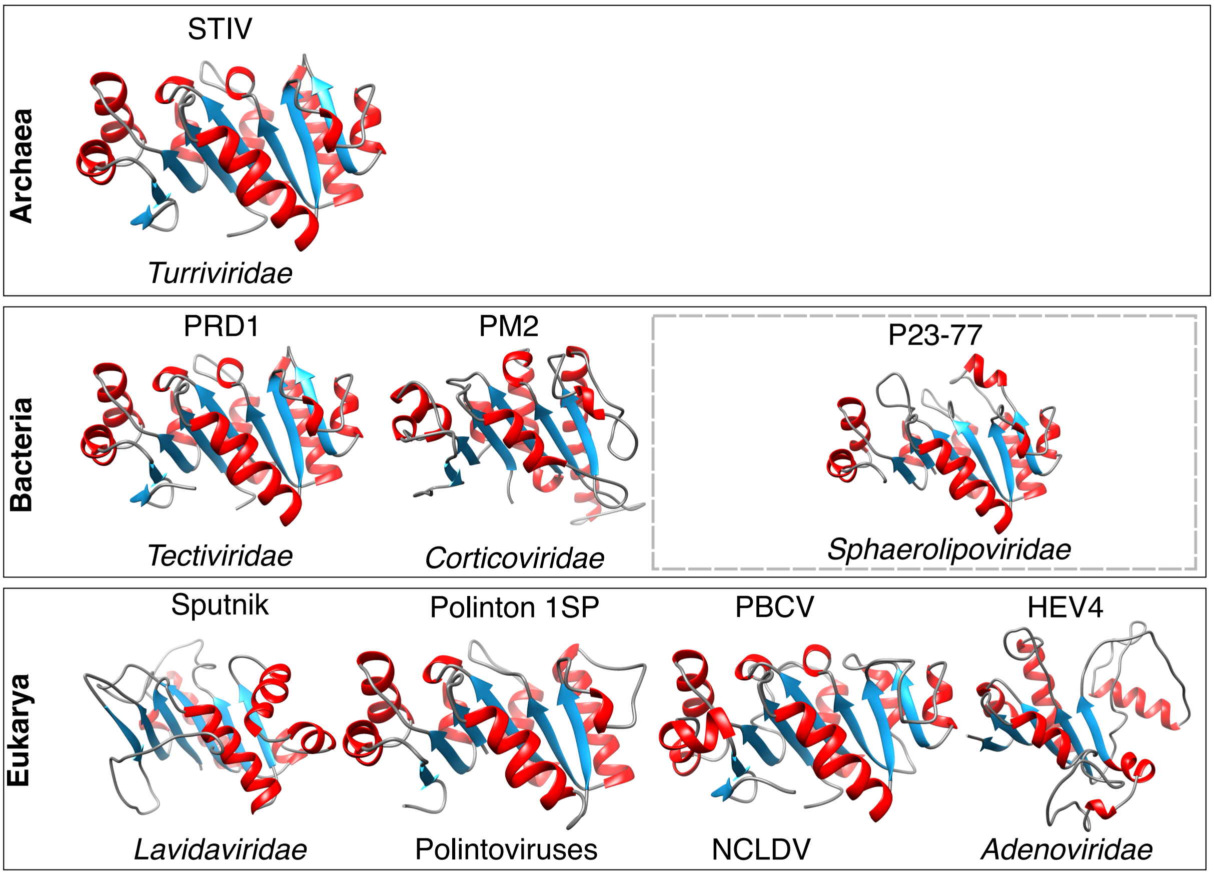

Supplementary Figure 2. Packaging ATPases of the *Varidnaviria* realm. Packaging ATPases (pATPases) are arranged horizontally according to the domain of life to which their hosts are classified. Virus names are provided above the structural models of various families of bacterial, archaeal and eukaryotic viruses. The structures are coloured according to the secondary structure elements: α-helices in red; β-strands in blue; and random coil in grey. The X-ray structures of the pATPase proteins of Sputnik, Polinton 1 SP, PBCV, HAE4, PRD1, PM2 and P23-77 are not available and are represented with homology-based models. Sputnik, Sputnik virophage; Polinton 1 SP, Polinton 1 Strongylocentrotus purpuratus; PBCV, Paramecium bursaria Chlorella virus; NCLDV, nucleo-cytoplasmic large DNA viruses; HAE4, Human adenovirus E4; PRD1, Enterobacteria phage PRD1; PM2, Pseudoalteromonas phage PM2; STIV, Sulfolobus turreted icosahedral virus. P23-77, Thermus virus P23-77. RCSB Protein Data Bank (PDB) accession numbers for the pATPase structure: STIV, PDB entry 4KFU.

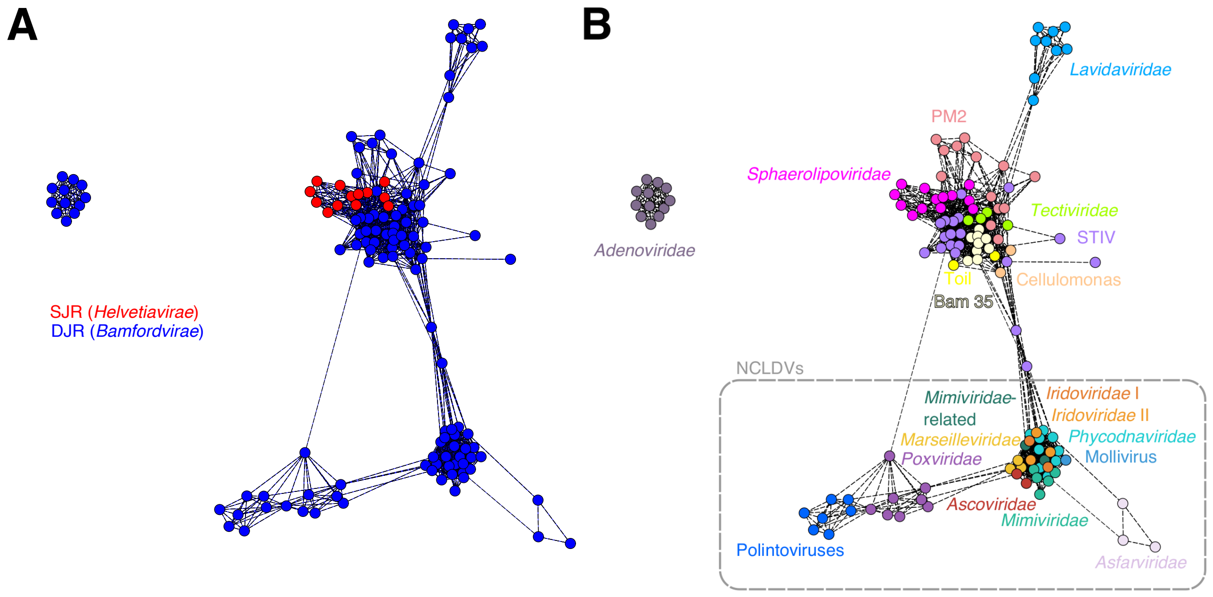

Supplementary Figure 3. Sequence similarity networks of the packaging ATPase proteins from the *Varidnaviria* realm. Protein sequences were clustered using SiLiX based on the sequence similarity. Different clusters of viruses are shown as clouds of connected colored circles, with each circle corresponding to a single pATPase protein. Members are classified according to (**A**) the structure of the MCP/kingdom or (**B**) the different families/subfamilies which they belong to. Abbreviations: DJR, double jelly-roll; SJR: single jelly-roll; NCLDV, Nucleo-Cytoplasmic Large DNA Viruses; STIV, Sulfolobus turreted icosahedral virus

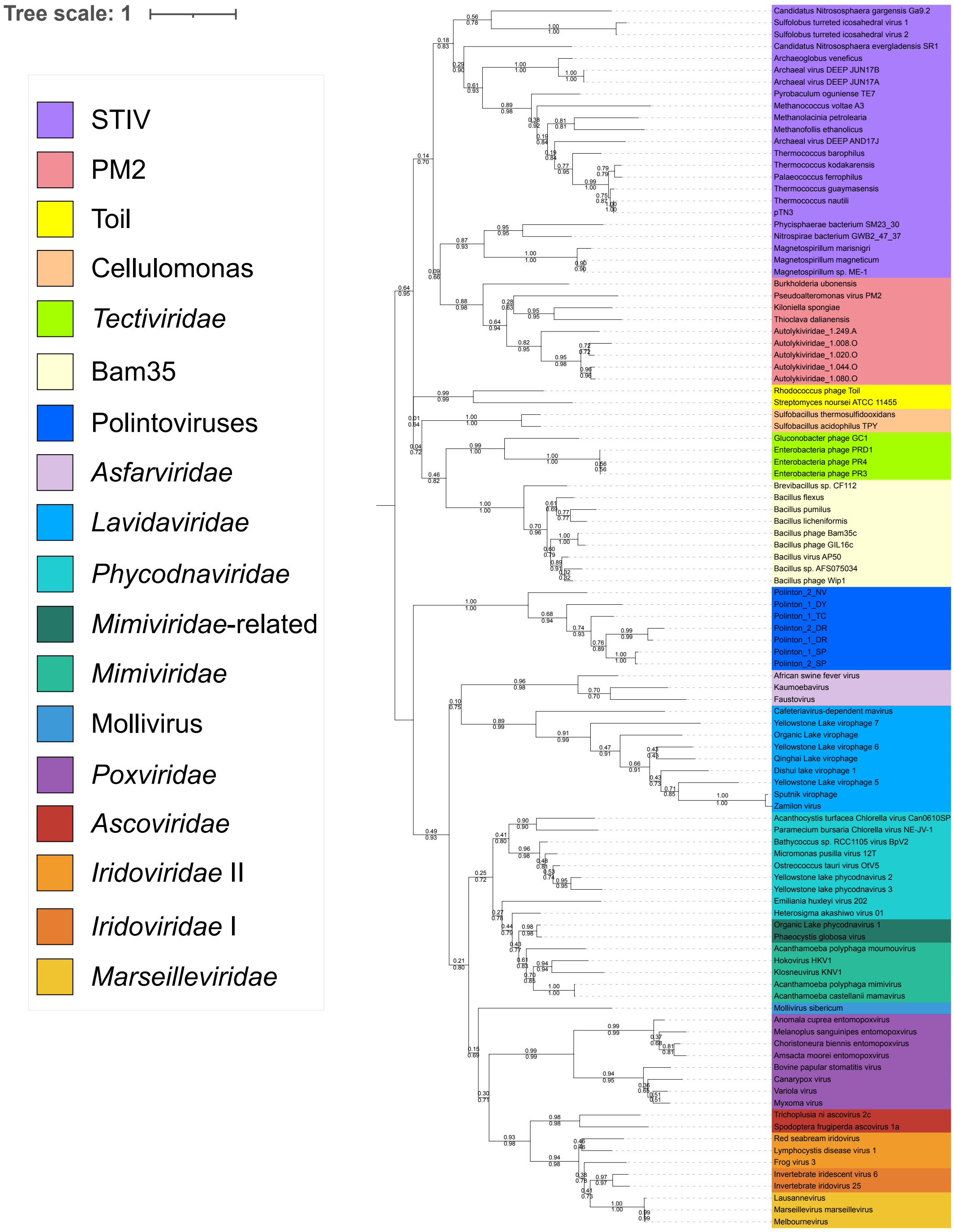

Supplementary Figure 4. Phylogenetic tree of the major capsid protein gene of the *Varidnaviria* realm, excluding *Adenoviridae, Sphaerolipoviridae*, Odin and FLiP members. The alignment has 103 sequences with 237 positions. The root of the phylogenetic tree was between the prokaryotic and eukaryotic members. The scale-bar indicates the average number of substitutions per site. The best-fit model was LG + F + R4, which was chosen according to Bayesian Information Criterion (BIC). Values on top and below branches represent support calculated by classical bootstrap and transfer bootstrap expectation (TBE) using 1000 replicates, respectively.

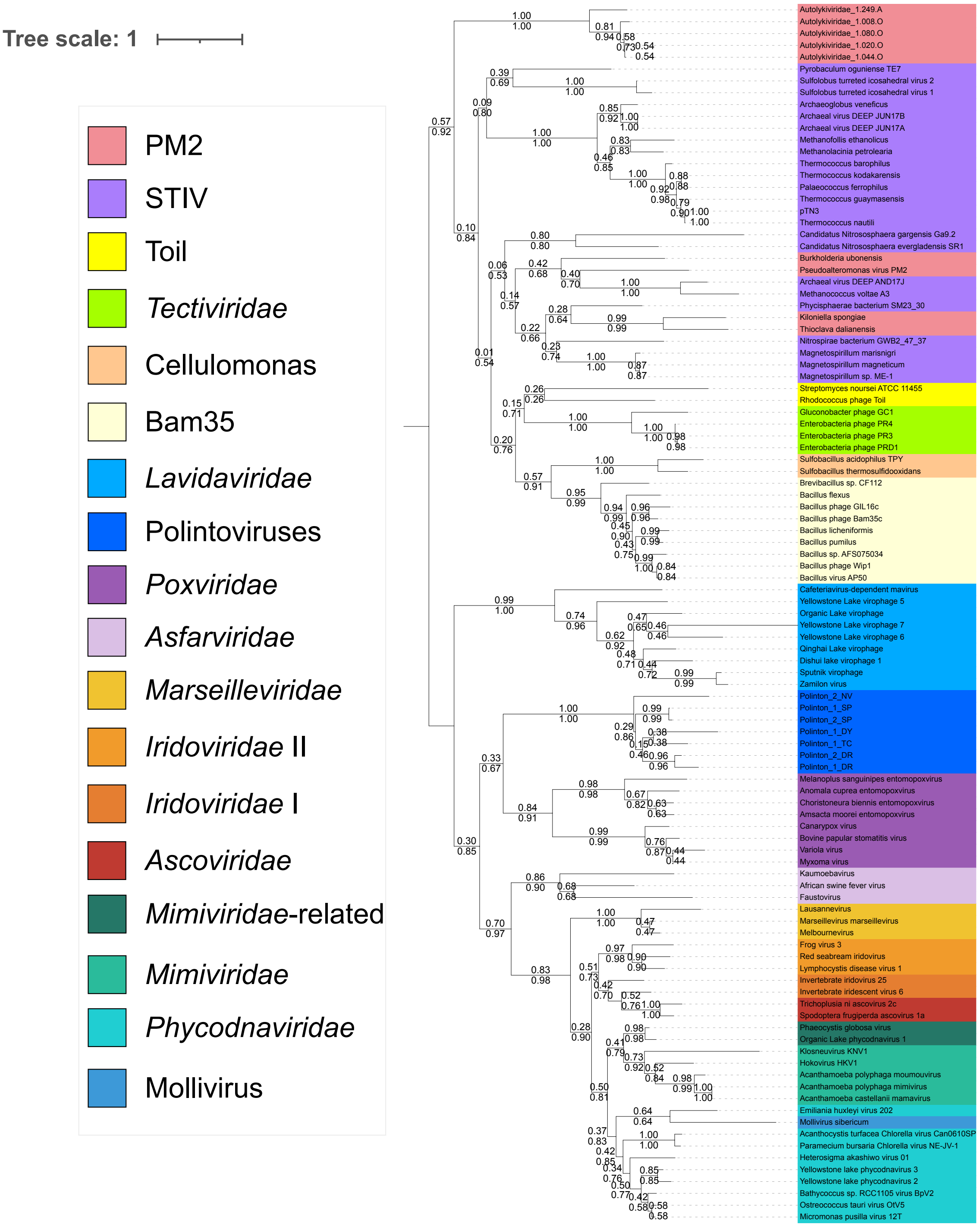

Supplementary Figure 5. Phylogenetic tree of the packaging ATPase gene of the double jelly-roll viruses from the *Varidnaviria* realm, excluding *Adenoviridae* and *Sphaerolipoviridae*. The alignment has 103 sequences with 171 positions. The root of the phylogenetic tree was between the prokaryotic and eukaryotic members. The scale-bar indicates the average number of substitutions per site. The best-fit model was LG + F+ R6, which was chosen according to Bayesian Information Criterion (BIC). Values on top and below branches represent support calculated by classical bootstrap and transfer bootstrap expectation (TBE) using 1000 replicates, respectively.

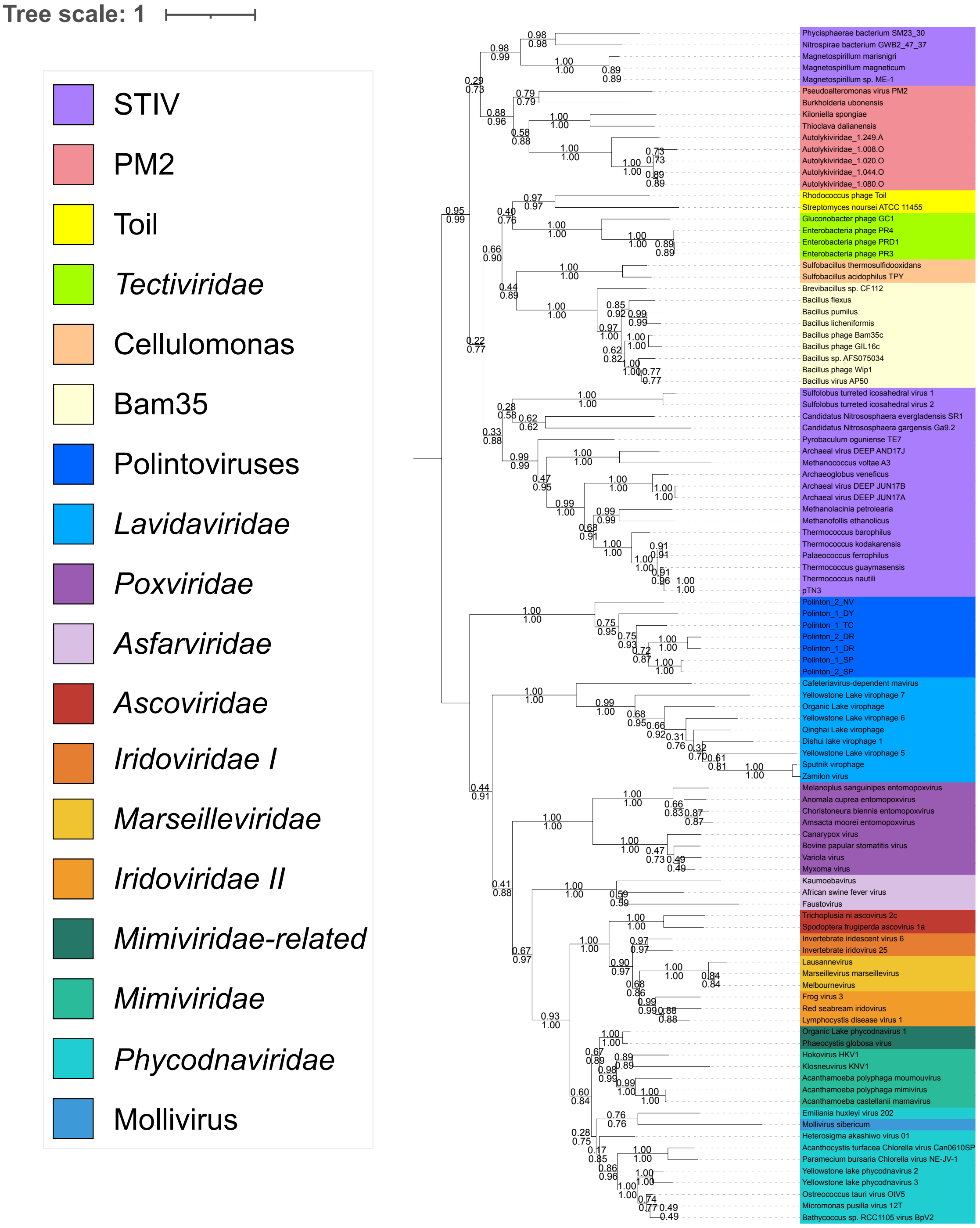

Supplementary Figure 6. Phylogenetic tree of the concatenated MCP and pATPase genes. Phylogenetic tree annotated with the corresponding group. The alignment has 103 sequences with 408 positions. Phylogenetic tree was rooted between the prokaryotic and eukaryotic members. The scale-bar indicates the average number of substitutions per site. The best-fit model was LG + F + R6, which was chosen according to Bayesian Information Criterion (BIC). Values on top and below branches represent support calculated by classical bootstrap and transfer bootstrap expectation (TBE) using 1000 replicates, respectively.

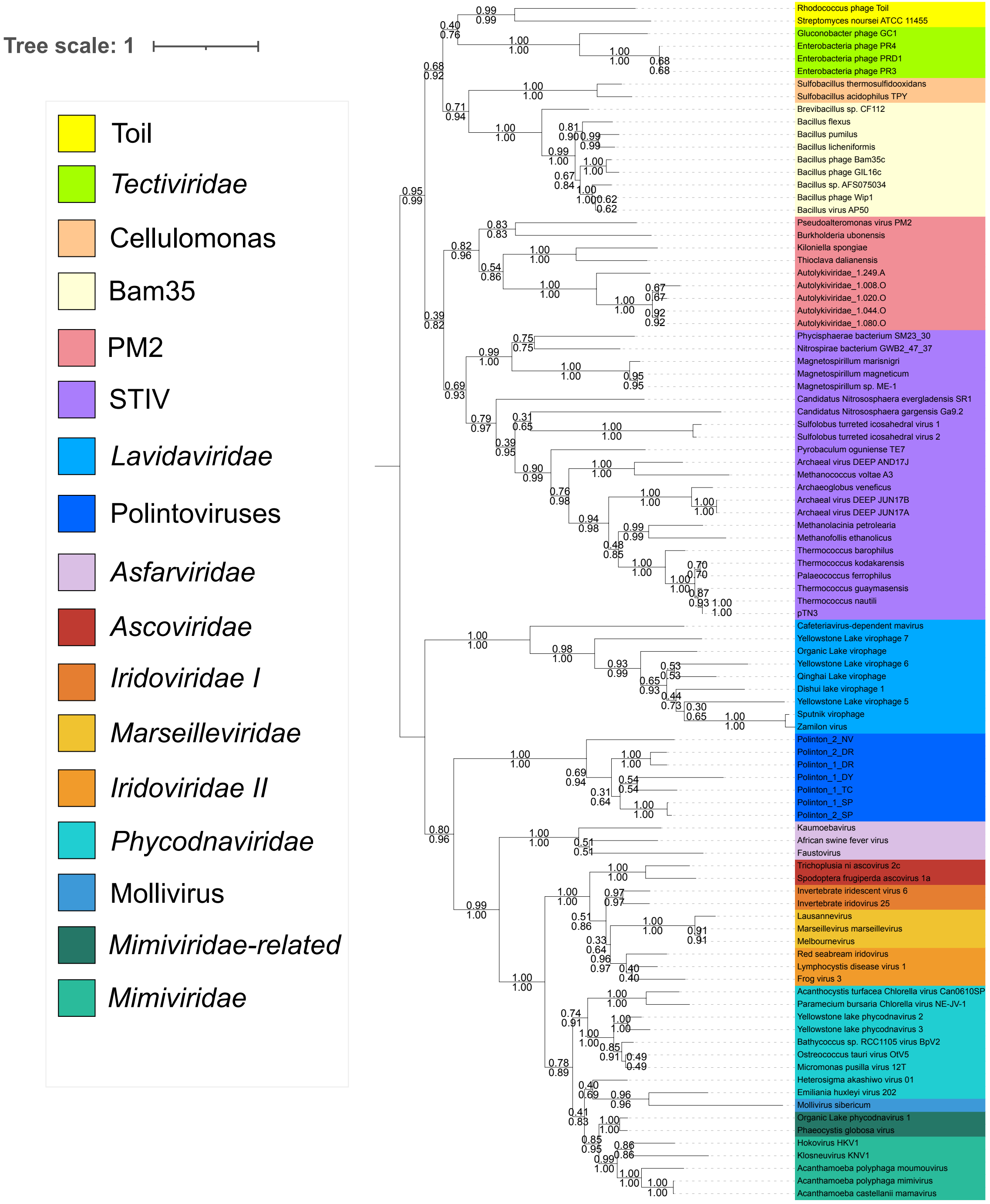

Supplementary Figure 7. Phylogenetic tree of the concatenated major capsid protein and packaging ATPase genes, excluding the *Poxviridae*. The alignment has 95 sequences with 434 positions. Phylogenetic tree was rooted between the prokaryotic and eukaryotic members. The scale-bar indicates the average number of substitutions per site. The best-fit model was LG + F + R5, which was chosen according to Bayesian Information Criterion (BIC). Values on top and below branches represent support calculated by classical bootstrap and transfer bootstrap expectation (TBE) using 1000 replicates, respectively.

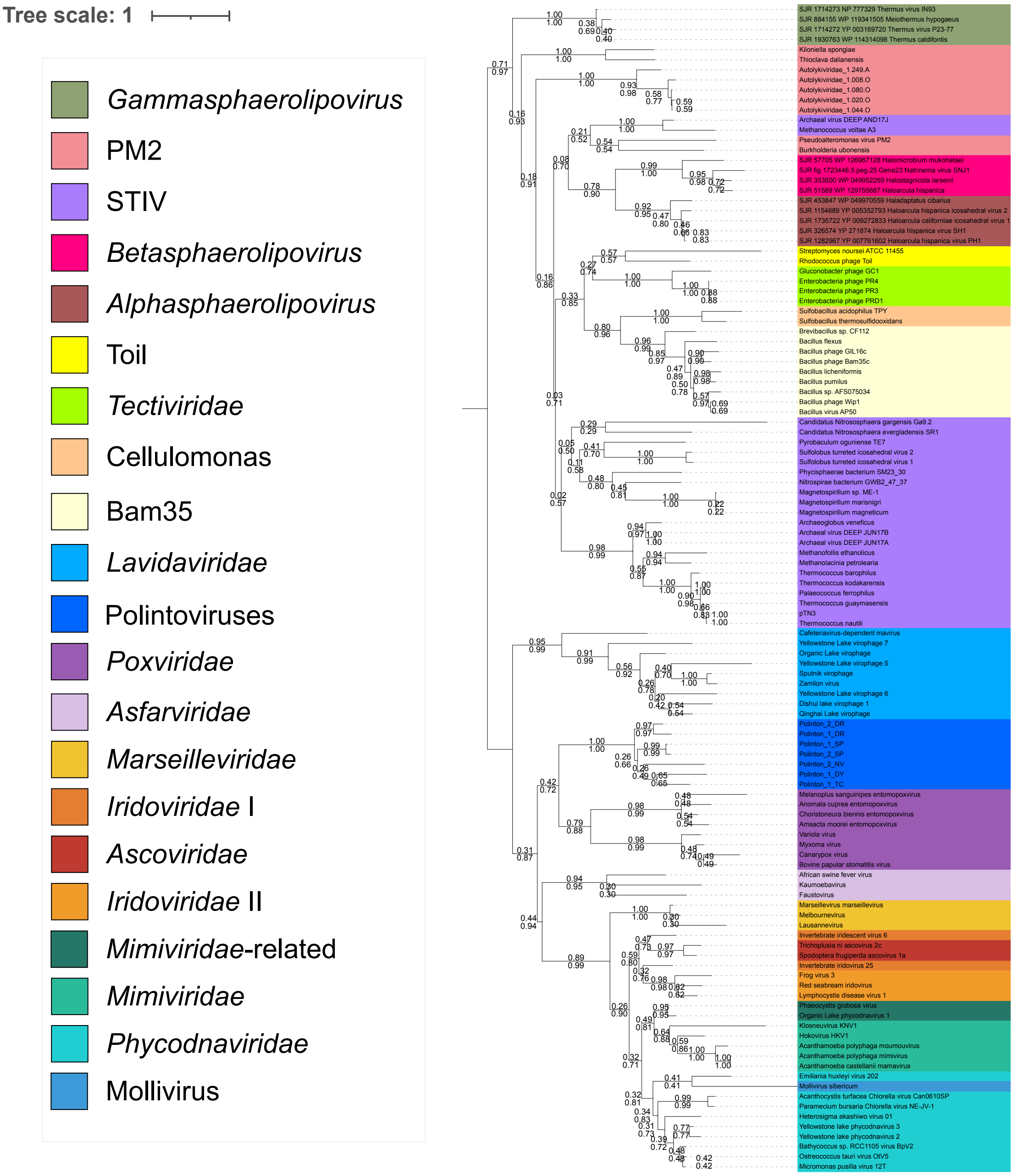

Supplementary Figure 8. Phylogenetic tree of the packaging ATPase gene of the *Varidnaviria* realm, excluding *Adenoviridae.* The alignment has 116 sequences with 151 positions. The root of the phylogenetic tree was between the prokaryotic and eukaryotic members. The scale-bar indicates the average number of substitutions per site. The best-fit model was LG + R6, which was chosen according to Bayesian Information Criterion (BIC). Values on top and below branches represent support calculated by classical bootstrap and transfer bootstrap expectation (TBE) using 1000 replicates, respectively.
